# Monoterpenoids (thymol, carvacrol and S-(+)-linalool) with anesthetic activity in silver catfish (*Rhamdia quelen*): evaluation of acetylcholinesterase and GABAergic activity

**DOI:** 10.1590/1414-431X20176346

**Published:** 2017-10-19

**Authors:** A.E. Bianchini, Q.I. Garlet, J.A. da Cunha, G. Bandeira, I.C.M. Brusque, J. Salbego, B.M. Heinzmann, B. Baldisserotto

**Affiliations:** 1 Programa de Pós-Graduação em Farmacologia, Universidade Federal de Santa Maria, Santa Maria, RS, Brasil; 2 Curso de Medicina Veterinária, Universidade Federal de Santa Maria, Santa Maria, RS, Brasil

**Keywords:** AChE, Anesthesia, Benzodiazepine, Fish, GABA_A_, Terpenoids

## Abstract

This study evaluated the anesthetic potential of thymol and carvacrol, and their influence on acetylcholinesterase (AChE) activity in the muscle and brain of silver catfish (*Rhamdia quelen*). The AChE activity of S-(+)-linalool was also evaluated. We subsequently assessed the effects of thymol and S-(+)-linalool on the GABAergic system. Fish were exposed to thymol and carvacrol (25, 50, 75, and 100 mg/L) to evaluate time for anesthesia and recovery. Both compounds induced sedation at 25 mg/L and anesthesia with 50–100 mg/L. However, fish exposed to carvacrol presented strong muscle contractions and mortality. AChE activity was increased in the brain of fish at 50 mg/L carvacrol and 100 mg/L thymol, and decreased in the muscle at 100 mg/L carvacrol. S-(+)-linalool did not alter AChE activity. Anesthesia with thymol was reversed by exposure to picrotoxin (GABA_A_ antagonist), similar to the positive control propofol, but was not reversed by flumazenil (antagonist of benzodiazepine binding site), as observed for the positive control diazepam. Picrotoxin did not reverse the effect of S-(+)-linalool. Thymol exposure at 50 mg/L is more suitable than carvacrol for anesthesia in silver catfish, because this concentration did not cause any mortality or interference with AChE activity. Thymol interacted with GABA_A_ receptors, but not with the GABA_A_/benzodiazepine site. In contrast, S-(+)-linalool did not act in GABA_A_ receptors in silver catfish.

## Introduction

Anesthetics have many applications in aquaculture, used to improve animal welfare during and after management practices, during transport, and also for surgery. The most commonly used anesthetics for fish are tricaine methanesulfonate (MS-222), benzocaine, metomidate, 2-phenoxyethanol and quinaldine. However, undesirable effects due to the use of these anesthetics have been reported (1). Studies have investigated the use of essential oils (EOs) from plants and their isolated compounds with the objective of finding new safe and effective anesthetics with fewer side effects (2,3).

One important characteristic of EOs related to their effectivity as anesthetics is their lipophilic properties, as high lipid solubility contributes to rapid diffusion through biological membranes (1). Furthermore, EOs are primarily made up of terpenoids, substances whose pharmacological activities on the central nervous system (CNS) are frequently described. Such activities are connected with the ability of terpenes to cross the blood-brain barrier, where they modulate brain function (4).

**Figure 1.**
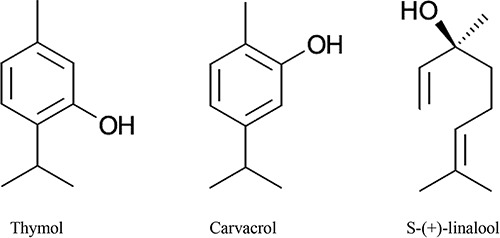
Chemical structures of the evaluated monoterpenoids.

Monoterpenoids (C_10_) are the predominant constituents in EOs (5). Among these, thymol and carvacrol isomers (Figure 1) are found in the EOs of plants including *Origanum vulgare* (6) and *Lippia sidoides* (7). Both isomeric forms have demonstrated anticonvulsant activity in mice, mediated by GABA (γ-aminobutyric acid) receptor activity, the main inhibitory neurotransmitter in the brain (8). These data suggest that there may be a possible sedative and/or anesthetic activity of thymol and carvacrol in fish via a mechanism similar to that reported in rodents, as modulation of GABA_A_ is frequently involved in the mechanism of action of anesthetics. For example, the anesthetic propofol is a widely used GABA_A_ agonist for inducing anesthesia in human and veterinary medicine, which also has proven effects in fish (9). Furthermore, EOs of *L. sidoides* containing the thymol and carvacrol chemotypes have been reported to act as anesthetics in silver catfish (*Rhamdia quelen*), which further strengthens this hypothesis (7).

Another monoterpenoid that deserves attention is linalool, as recent studies have suggested a potential anesthetic activity in fish (3,10). Linalool is present in EOs from aromatic plants in both enantiomeric forms, such as EOs derived from *Ocimum basilicum* L. (69.54% R-(-)-linalool) (11) and *Lippia alba* (59.66% S-(+)-linalool, Figure 1) (12). However, the biological activities and mechanism of action of S-(+)-linalool are poorly understood. S-(+)-linalool acts as a sedative and anesthetic in silver catfish, but these activities are not associated with the benzodiazepine site of GABA_A_ receptors (10). No studies have investigated whether S-(+)-linalool interacts with other GABA_A_ sites, which has encouraged further investigation.

Some monoterpenoids can positively or negatively modulate the enzymatic activity of acetylcholinesterase (AChE), the enzyme responsible for hydrolyzing the neurotransmitter acetylcholine (ACh) (13). In fish, inhibition of AChE results in the accumulation of ACh in nervous terminations, and consequently, hyperstimulation of muscarinic and nicotinic receptors, which can cause muscle contractions and erratic swimming, in addition to impaired feeding and reproduction (14). Therefore, studies evaluating AChE activity in fish exposed to these compounds are useful for identifying any potential negative effects associated with the modulation of AChE activity. The brain AChE activity was not affected in silver catfish sedated and/or anesthetized with the essential oil of *Aloysia gratissima* (2) and methanolic extract of *Condalia buxifolia* (15).

The objectives of this study were to evaluate the anesthetic potential of thymol and carvacrol, their action on GABA_A_ receptors, and their influence on the activity of AChE in silver catfish. We also investigated the mechanism of action of the anesthetic effect of S-(+)- linalool on GABA_A_ receptors and the influence on AChE activity.

## Material and Methods

### Animals

Silver catfish juveniles were obtained from a fish farm in Cruz Alta (Southern Brazil) and transported to the Fish Physiology Laboratory at the Universidade Federal de Santa Maria (UFSM). Fish were allowed to acclimatize for 1 week in continuously aerated 250 L tanks prior to the experiments. Dissolved oxygen and temperature (oximeter 550A; YSI, USA), pH (microprocessor pH meter, AT-315; Alfakit, Brazil) and total ammonia nitrogen (TAN) (16) were monitored daily, and maintained within the recommended values for this species (temperature 19.76±0.11°C, pH 7.15±0.12, dissolved oxygen 6.86±0.33 mg/L, and TAN 0.12±0.02 mg N/L). Animals were fed to satiation with commercial feed (42% crude protein, Supra-Alisul Alimentos®, Brazil) once per day, and fasted for 24 h before the experiments. Each animal was used only once. The protocol was approved by the Ethics and Animal Welfare Committee of UFSM (process No. 074/2014).

### Essential oil (EO) extraction and S-(+)-linalool isolation

S-(+)-linalool (density: 0.85 g/mL) was isolated from the EO of *Lippia alba*, which was cultivated in the UFSM campus in Frederico Westphalen, Brazil (Voucher No. 10050, Department of Biology, UFSM). The EO extraction was performed by steam distillation of fresh leaves, using a modified Clevenger apparatus for 3 h, according to the method described by the European Pharmacopoeia (17). The EO was stored in a sealed amber glass vial at –4°C until the isolation process. The isolation of S-(+)-linalool from EO was performed using column chromatography. Compound identification and purity was evaluated by gas chromatography-mass spectrometry, as described by Heldwein et al. (10).

### Commercially obtained drugs

The thymol (density: 0.965 g/mL) and carvacrol (density: 0.977 g/mL) isomers (≥99.0 purity) used for the anesthetic induction tests were purchased from Sigma-Aldrich, Brazil. Picrotoxin (Sigma-Aldrich), a blocker of the GABA_A_ receptor type, and propofol (Propotil®, Biochimico, Brazil), a GABA_A_ agonist anesthetic, were included in the protocol in order to evaluate the sedative and anesthetic effects of the studied compounds on the GABA_A_ receptor type. Diazepam (Uni-Diazepax®, Chemical Union, Brazil) and flumazenil (Flamazil®, Cristália Ltda., Brazil), selective agonist and antagonist, respectively, of the benzodiazepine site of the GABA_A_ receptor, were used to evaluate the anesthetic effect of thymol on benzodiazepine receptors (18). Prior to the experiments, S-(+)-linalool, carvacrol and thymol were diluted in 95% ethanol (1:10), and picrotoxin was diluted in Tween 80 (0.033% in water). Propofol, diazepam and flumazenil have good solubility in water and did not require solubilization prior to the experiments.

### Experiment 1: Induction of anesthesia and recovery

Silver catfish juveniles (n=6 for each concentration and compound; 12.84±0.34 g and 10.5±0.22 cm) were individually exposed to 25, 50, 75, or 100 mg/L thymol or carvacrol in an aquarium filled with 1 L of water. The anesthetic induction stages were evaluated as described by Gomes et al. (19): stage (S) 2, sedation characterized by the loss of response to external stimuli, determined by hitting the bottom of the aquarium with a glass rod; S3a, characterized by a total loss of balance, with fish able to maintain their swimming ability; S3b, total loss of balance and swimming ability, with fish still responsive to application of pressure on the caudal peduncle using a glass rod; and S4, deep anesthesia, in which fish do not respond to any external stimuli. Evaluation was carried out until the animals reached the anesthesia stage, or after a maximum period of 30 min. Fish were then placed in anesthetic-free aquaria until complete recovery, or a maximum period of 30 min. The animals were considered recovered once they had demonstrated normal swimming capacity and equilibrium, and also responded to stimulation from a glass rod hitting the aquarium bottom. Survival of the animals was assessed 48 h after the experiment.

### Experiment 2: AChE activity

As muscle contractions were observed in experiment 1, it was necessary to evaluate the AChE activity in the brain and muscle of silver catfish exposed to monoterpenoid with anesthetic properties used in the studies.

Silver catfish juveniles (n=6 for each concentration and compound; 9.51±0.32 g and 10.0±0.25 cm) were exposed to 50 or 100 mg/L of thymol or carvacrol and 153 mg/L of S-(+)-linalool for 10 min (mean time for anesthesia). These fish were then euthanized by spinal cord section to collect the brain and muscle. Samples from the control group (exposed to water only) and from fish exposed to the highest ethanol concentration (1377 mg/L; used to dilute the compounds) were also collected. Enzymatic activity was determined by the Ellman et al. (20) method, with modifications. Protein concentration was determined by the Coomassie Blue method (21). The enzyme activity is reported as µmol of acetylthiocholine (ASCh) hydrolyzed per milligram of protein per minute.

### Experiment 3: Evaluation of activity on GABAA receptors

Silver catfish juveniles (8.86±0.42 g and 9.47±0.17 cm) were exposed to S-(+)-linalool, thymol or propofol (n=16 per group) at their respective anesthetic (S4) concentrations (153, 50 and 2.5 mg/L). The mechanism of action of carvacrol was not investigated because it did not show good performance as an anesthetic (see results). After induction of anesthesia, the animals were transferred to aquaria with anesthetic-free water (n=8) or to aquaria containing 100 mg/L picrotoxin (n=8) to measure their partial recovery (response of fish to pressure stimulus on the caudal peduncle with a glass rod) and total recovery (normal swimming with response to external stimulus) time. The maximum observation time was 30 min. The concentrations of propofol and S-(+)-linalool used in this experiment were selected according to Gressler et al. (9) and Heldwein et al. (10), respectively, and the choice of thymol and picrotoxin concentrations were in accordance with preliminary tests.

To ensure that picrotoxin itself did not present a stimulatory effect, silver catfish (n=4) were exposed to picrotoxin (100 mg/L) or water for 30 min. No distinct behavioral differences were observed between groups.

### Experiment 4: Evaluation of activity on the benzodiazepine site of GABAA receptors

This experiment evaluated the involvement of the benzodiazepine site on GABA_A_ receptors in the anesthetic effect of thymol. Fish (11.63±0.42 g and 9.85±0.10 cm) were anesthetized with 50 mg/L thymol or 42 mg/L diazepam (n=16 per group). Fish were then placed in an anesthetic-free aquarium (n=8) or in an aquarium containing 3.0 mg/L flumazenil (n=8) to recover. The partial and total recovery times for each animal were evaluated as described in experiment 3. The concentrations of diazepam and flumazenil used in this experiment were selected according to Garlet et al. (22).

### Statistical analysis

The data were submitted to Levene's test to determine homogeneity of variances, then one- or two-way ANOVA was performed, and where appropriate, followed by Tukey's *post hoc* test. Kruskal-Wallis ANOVA by ranks was used for nonparametric data obtained for brain AChE activity (thymol 50 and 100 mg/L). The tests were performed using Statistica software (version 11.0), and the minimum significance level for all analyses was 5% (P<0.05). Data are reported as means±SE.

## Results

### Experiment 1: Induction of anesthesia and recovery

No mortality was detected 48 h after exposure to thymol, whereas carvacrol caused 50, 33, 33, and 16% mortality during the first 24 h after exposure to 25, 50, 75, and 100 mg/L, respectively. Involuntary muscle contractions were observed in animals exposed to all concentrations of carvacrol. The same was observed for exposure to all concentrations of thymol, but with a reduced frequency and intensity when compared to carvacrol.

Regression analysis showed a concentration-response relationship for thymol and carvacrol for all stages of anesthetic induction, but not for recovery (Table 1). Carvacrol induced S2 (all concentrations), S3a and S3b (25 and 50 mg/L) stages faster than thymol. However, exposure to thymol at 100 mg/L induced deep anesthesia (S4) significantly faster than carvacrol. Only fish exposed to 50 mg/L carvacrol recovered within 30 min, and the recovery time was significantly longer than recovery from anesthesia with the same concentration of thymol. On the other hand, fish anesthetized with 100 mg/L thymol did not completely recover within 30 min (Table 1).

**Table 1.** Time (in s) required for induction and recovery from anesthesia with thymol and carvacrol in silver catfish (*Rhamdia quelen)* juveniles.

Concentration (mg/L)	Stage (time until induction, s)				
	S2	S3a	S3b	S4	Recovery
Thymol					
25	109.66 ± 2.23	319.66 ± 46.23	611.00 ± 37.65	>1800	1649.00 ± 47.78
50	55.66 ± 3.68	86.67 ± 6.78	284.00 ± 23.96	491.50 ± 10.49	919.67 ± 122.55
75	20.00 ± 1.41	40.83 ± 5.13	175.00 ± 14.68	373.33 ± 15.85	1375.67 ± 150.86
100	14.33 ± 2.40	28.50 ± 2.84	55.80 ± 7.16	170.00 ± 11.87	>1800
Equation	y=190.75 – 3.70x+ 0.0193x2R2=0.98	y=624.58 – 14.71x+ 0.0883x2R2=0.82	y=1384.20 – 42.89x+ 0.5395x2 – 0.0024x3R2=0.94	y=472.33 + 3.79x – 0.0681x2R2=0.95	
Carvacrol					
25	58.83 ± 1.85*	74.00 ± 1.73 *	352.33 ± 22.56*	>1800	>1800
50	33.50 ± 2.86*	51.33 ± 3.29 *	76.75 ± 2.72*	524.67 ± 36.09	1580.83 ± 81.69*
75	12.33 ± 0.88*	30.83 ± 1.94	195.00 ± 11.05	386.33 ± 9.26	>1800
100	8.67 ± 0.88*	24.67 ± 1.33	70.17 ± 3.61	299.17 ± 13.42*	>1800
Equation	y=98.33 – 1.77x+ 0.0087x2R2=0.95	y=75.00 – 0.29x – 0.018x2 + 0.0001x^3^R2=0.96	y=1655.16 – 81.14x+ 1.33x2 – 0.0068x3R2=0.95	y=954.83 – 10.65x+ 0.0409x2R2=0.77	

In the equations, x represents the concentration of the compound (mg/L), and y represents the time taken to reach the stage of induction or recovery from anesthesia (s). Data are reported as means±SD (n=6).

* P<0.05, compared to thymol in the same concentration and induction stage (one-way ANOVA and Tukey's test).

### Experiment 2: AChE activity

**Figure 2.**
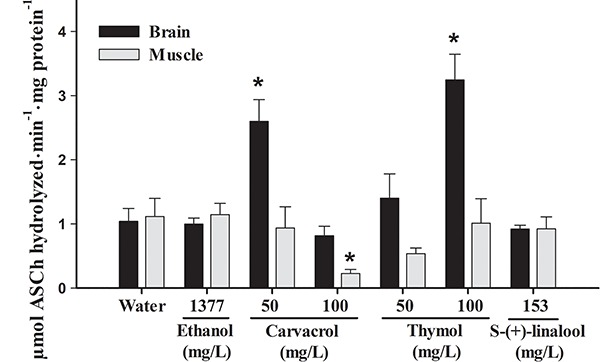
Acetylcholinesterase (AChE) activity in the brain and muscle of silver catfish (*Rhamdia quelen*) juveniles anesthetized with thymol and carvacrol (50 and 100 mg/L) and S-(+)-linalool (153 mg/L). Data are reported as means±SD (n=6). ASCh: acetylthiocholine. *P<0.05, compared to the control (water) group in the same tissue (one-way ANOVA and Tukey's test or Kruskal-Wallis ANOVA by ranks).

Exposure to ethanol and S-(+)-linalool (153 mg/L) did not significantly alter AChE activity in both tissues (muscle and brain) compared to the control group. Fish anesthetized with 50 mg/L carvacrol and 100 mg/L thymol showed significantly higher brain AChE activity compared to the control group. In the muscle, only 100 mg/L carvacrol decreased AChE activity compared to the control group (Figure 2).

### Experiment 3: Evaluation of activity on GABAA receptors

**Figure 3.**
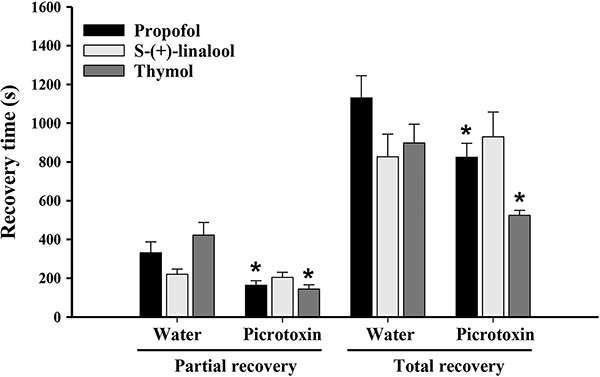
Time required for recovery (partial and total) in water and picrotoxin (100 mg/L) of silver catfish (*Rhamdia quelen*) juveniles anesthetized with propofol (2.5 mg/L), S-(+)-linalool (153 mg/L) and thymol (50 mg/L). Data are reported as means±SD (n=8). *P<0.05, compared to the group that recovered in water (two-way ANOVA and Tukey's test).

Partial and total recovery of animals anesthetized with propofol was significantly faster with picrotoxin than water, validating the protocol used. The same was observed for the partial and total recovery of fish exposed to thymol. Picrotoxin did not alter the recovery of fish anesthetized with S-(+)-linalool when compared to water control group (Figure 3).

### Experiment 4: Evaluation of activity on the benzodiazepine site of GABAA receptors

**Figure 4.**
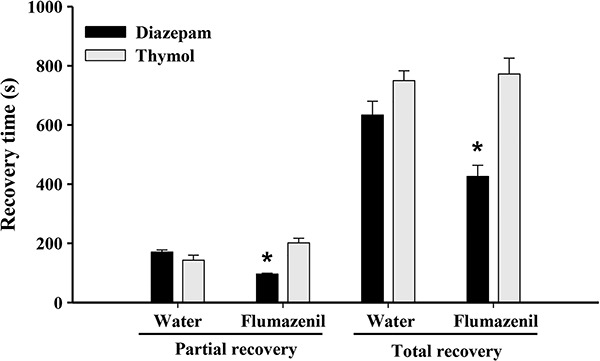
Time required for recovery (partial and total) in water and flumazenil (3 mg/L) of silver catfish (*Rhamdia quelen*) juveniles anesthetized with diazepam (42 mg/L) and thymol (50 mg/L). Data are reported as means±SD (n=8). *P<0.05, compared to the group that recovered in water (two-way ANOVA and Tukey's test).

Fish anesthetized with diazepam showed significantly faster partial and total recovery in the flumazenil recovery bath when compared with the water control group. However, the recovery of fish anesthetized with thymol was not affected by flumazenil (Figure 4).

## Discussion

### Anesthesia

Thymol and carvacrol were found to be sedatives (25 mg/L) and anesthetics (50-100 mg/L) in silver catfish. These results were expected, as the EOs of *L. sidoides* from the thymol and carvacrol chemotypes (containing 68.40 and 67.89% of these compounds, respectively) have been previously reported to induce anesthesia in silver catfish (7). In addition, Brito and Brito (23) reported the popular use of the EO of *L. sidoides* thymol chemotype as a local anesthetic and sedative. Moreover, recent studies with rodents have reported the anxiolytic and antidepressant action of carvacrol (24,25) and anticonvulsant activity of thymol (26).

Anesthesia was induced in silver catfish with 50 mg/L thymol and carvacrol, and the time of induction and recovery was lower when compared to other terpenoids, such as (+)-dehydrofukinone (50 mg/L) and (+)-spathulenol (51.2 mg/L), as evaluated in the same species (2,22). Thymol and carvacrol were found to be more potent than S-(+)- linalool, as only 50 mg/L of both compounds was required to induce the S4 stage, whereas fish exposed to 51 mg/L S-(+)-linalool had previously been reported to only reach the S3a stage (10). In common carp (*Cyprinus carpio*), terpenoids such as menthol, myrcene and linalool (enantiomeric form not specified) were also reported to act as anesthetics at concentrations ≥50 mg/L (50, 150, and 200 mg/L, respectively) (3,27).

Exposure to 25 mg/L thymol for 30 min only induced sedation, indicating that this concentration (or lower) may be suitable for use in fish transport. The sedation of fish prior to transportation is carried out to reduce stress and physiological responses that are detrimental to animal welfare (15). For anesthesia, 100 mg/L thymol has been reported to induce anesthesia in less than 3 minutes (the optimal time for anesthesia according to Gilderhus and Marking) (28), however, the total recovery exceeded 30 min. Prolonged recovery has also been reported for other anesthetics (22), and may be related to slow clearance. Therefore, intermediate concentrations (50 and 75 mg/L) have a higher cost-benefit rate as they are associated with faster recovery.

Compared to thymol, carvacrol induced sedation in a shorter time, especially at concentrations of 25 and 50 mg/L. However, the side effects of carvacrol observed during (strong muscular contractions) and after anesthesia (mortality) and long recovery time prevent its application as an anesthetic. These results corroborate those of Silva et al. (7) in silver catfish anesthetized with the EO of *L. sidoides* containing thymol and carvacrol. However, the muscle contractions occurred independent of the concentration used, and therefore, they are not a determining factor for carvacrol mortality. Furthermore, there was no mortality associated with thymol exposure, despite fish presenting mild contractions.

Interestingly, the mortality induced by carvacrol exposure decreased with increasing concentrations. At lower concentrations, fish required a longer period of carvacrol exposure to reach the desired stage, which may have contributed to the increased mortality observed. The long exposure period, combined with the high lipid solubility of anesthetics, facilitates accumulation in lipophilic compartments such as biological membranes and adipose tissue, thereby hindering their elimination (1).

### AChE activity

The observation of muscle contractions in fish is attributed to the anticholinesterase activity of some compounds (14). However, in this study, only 100 mg/L carvacrol was found to reduce AChE activity in muscle, contrary to what was expected, as muscle contractions were observed during anesthesia at all concentrations tested. Thymol, which also caused muscle contractions but with reduced intensity and frequency than carvacrol, did not inhibit AChE activity at any concentration tested. Therefore, it appears that the muscle contractions induced by thymol and carvacrol involve a mechanism unrelated to AChE inhibition. Studies in the literature have reported inhibition of AChE activity *in vitro* by thymol and carvacrol, however, the activity of carvacrol was found to be 10-fold more potent than thymol (29). This difference in potency for inhibition of the enzyme could explain why thymol did not inhibit AChE activity *in vivo* at the concentrations tested. Consequently, thymol may be a safer anesthetic than carvacrol. Similarly, our observation that exposure to S-(+)-linalool at an anesthetic concentration did not alter AChE activity emphasizes its efficacy as an anesthetic.

Unlike the muscle, AChE activity in the brain was increased in silver catfish exposed to 50 and 100 mg/L of carvacrol and thymol, respectively. This result is rather curious, although similar results have been reported for other compounds. A study by López et al. (13) showed that *in vitro* administration of compounds of the same class, such as γ-terminene, geraniol and camphor, activated AChE at a lower concentration (0.04 mM), whereas a higher concentration (5 mM) caused inhibition. Therefore, we can infer that the influence of monoterpenoids on AChE activity is likely to be concentration- and tissue-dependent.

### Mechanism of action for thymol and S-(+)-linalool

GABA_A_ receptors are ligand-regulated ion channels responsible for mediating rapid inhibitory synapses. Activation of GABA_A_ produces CNS depression, involved in the mechanism of action of anxiolytics, sedative-hypnotics, anticonvulsants and anesthetics (18). The interaction of thymol and S-(+)-linalool with GABA_A_ receptors was assessed in silver catfish by placing them in water containing picrotoxin (GABA_A_ receptor channel blocker) in order to evaluate recovery from sedation and/or anesthesia. Picrotoxin has convulsant properties, so it is only used for research purposes to induce seizures or antagonize the effect of GABA_A_ agonists (30).

Silver catfish that were anesthetized with thymol recovered faster in a picrotoxin bath when compared to the water control group. Likewise, the group anesthetized with propofol (positive control) also showed a reduced recovery time when placed in a picrotoxin bath. However, recovery from anesthesia induced by S-(+)-linalool was not altered by picrotoxin. The antagonistic effect of picrotoxin following thymol-induced anesthesia supports the proposed interaction of thymol with GABA_A_ receptors, but does not indicate a specific site of action within this receptor. The results observed for thymol in the current study corroborate those previously reported in *in vitro* studies (31,32).

Interaction with the GABA_A_ receptor can also occur through the GABA_A_/benzodiazepine binding site (18). These binding sites are known to modulate the affinity of the GABA_A_ receptor for GABA, increasing Cl^-^ influx through the channel (4). Interactions with the GABA_A_/benzodiazepine site have been previously evaluated for EOs and their isolated compounds with sedative and anesthetic activities in silver catfish (12,22). To assess the affinity of thymol for the GABA_A_/benzodiazepine binding site, we evaluated the effect of flumazenil, a competitive antagonist of this binding site (18). We did not observe any interaction between thymol and the GABA_A_/benzodiazepine site, as recovery from thymol-induced anesthesia was unaffected by flumazenil. This is in agreement with results from a study that evaluated human GABA_A_ receptors expressed in *Xenopus laevis* oocytes, in which the behavior of thymol was similar to a flumazenil-insensitive positive allosteric modulator at the GABA_A_/benzodiazepine site (33). Thymol also showed direct action (Cl^-^ currents induced in the absence of GABA) on the GABA_A_ receptors of HEK 293 cells, similar to the effect of propofol (34).

In contrast to results previously observed for thymol, our results did not indicate an interaction of S-(+)-linalool with GABA_A_ receptors. The interaction of S-(+)-linalool with the GABA_A_/benzodiazepine site in silver catfish had been disregarded in a previous study (10). Our results are similar to those reported by Silva Brum et al. (35), who suggested that the anticonvulsant activity of the racemic mixture of linalool did not result from interaction with GABA_A_ receptors. Instead, this compound is believed to interact with *N*-metil-D-aspartate (NMDA) receptors, although other mechanisms related to GABA release and absorption cannot be disregarded. Additionally, the antinociceptive activity of R-(-)-linalool in rodents has been attributed to its action on opioid, cholinergic (36), dopaminergic and glutamatergic systems (37). According to Leal-Cardoso et al. (38) the main mechanism by which linalool (enantiomeric form not specified) affects neuronal excitability is through the inhibition of voltage-regulated sodium channels and consequently blocking the action potentials.

The biological effects of linalool are related to different mechanisms of action, however, only Heldwein et al. (10) has reported the mechanism of action involved in the sedative and anesthetic activity for the enantiomer S-(+)-linalool to date. Other studies have described the effects of the racemic mixture, R-(-)-linalool, or did not specify the enantiomer used (39). It is noteworthy that the affinity and interaction with receptors, as well as the intensity of the biological effect, depends on the specificity of binding of the molecule to the receptor. Enantiomers may differ from each other in their pharmacodynamic and pharmacokinetic processes, therefore, the correct identification of these compounds is important (40).

In conclusion, thymol and carvacrol induced sedative and anesthetic activities in silver catfish at the same concentrations, however, carvacrol is not recommended as an anesthetic for fish due to the high rates of mortality after exposure. Thymol interacted with GABA_A_ receptors, but not with the GABA_A_/benzodiazepine site. In contrast, S-(+)-linalool did not appear to interact with the GABA_A_ receptors in fish.
